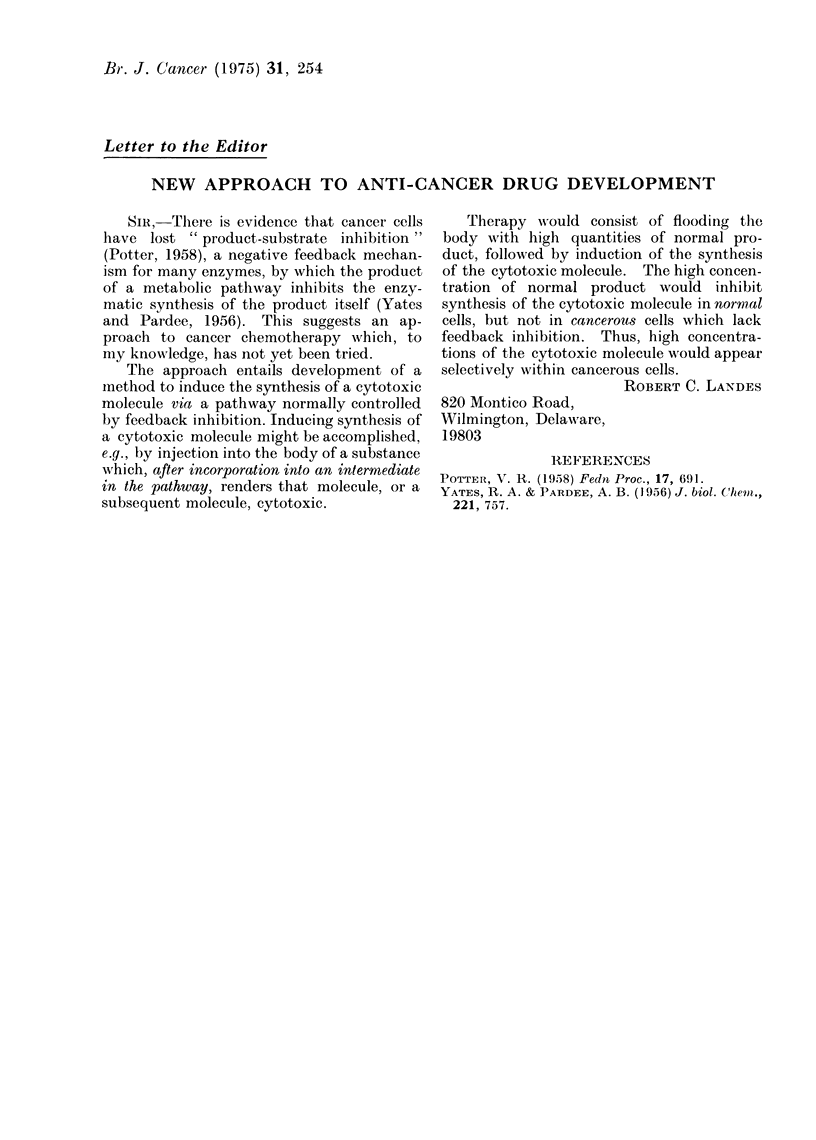# Letter: New approach to anti-cancer drug development.

**DOI:** 10.1038/bjc.1975.34

**Published:** 1975-02

**Authors:** R. C. Landes


*Br. J. Cancer* (1975) **31**, 254

### Letter to the Editor

### NEW APPROACH TO ANTI-CANCER DRUG DEVELOPMENT

SIR,—There is evidence that cancer cells have lost "product-substrate inhibition" (Potter, 1958), a negative feedback mechanism for many enzymes, by which the product of a metabolic pathway inhibits the enzymatic synthesis of the product itself (Yates and Pardee, 1956). This suggests an approach to cancer chemotherapy which, to my knowledge, has not yet been tried.

The approach entails development of a method to induce the synthesis of a cytotoxic molecule *via* a pathway normally controlled by feedback inhibition. Inducing synthesis of a cytotoxic molecule might be accomplished, *e.g.*, by injection into the body of a substance which, *after incorporation into an intermediate in the pathway*, renders that molecule, or a subsequent molecule, cytotoxic.

Therapy would consist of flooding the body with high quantities of normal product, followed by induction of the synthesis of the cytotoxic molecule. The high concentration of normal product would inhibit synthesis of the cytotoxic molecule in *normal* cells, but not in *cancerous* cells which lack feedback inhibition. Thus, high concentrations of the cytotoxic molecule would appear selectively within cancerous cells.

ROBERT C. LANDES
820 Montico Road,
Wilmington, Delaware,
19803

REFERENCES

POTTER, V. R. (1958) *Fedn Proc.*, **17**, 691.
YATES, R. A. & PARDEE, A. B. (1956) *J. biol. Chem.*, **221**, 757.